# Role of Bupropion and Naltrexone in Managing Depression With Polycystic Ovary Syndrome: A Case Report and Literature Review

**DOI:** 10.7759/cureus.11343

**Published:** 2020-11-05

**Authors:** Kaushal Shah, Ritu Kulkarni, Romil Singh, Harmandeep S Pannu, Dhwani Kamrai

**Affiliations:** 1 Psychiatry, Griffin Memorial Hospital, Norman, USA; 2 Psychology, University of Oklahoma, Norman, USA; 3 Internal Medicine, Metropolitan Hospital, Jaipur, IND; 4 Psychiatry and Behavioral Sciences, All Saints University School of Medicine, Roseau, DMA

**Keywords:** polycystic ovary syndrome (pcos), bupropion, naltrexone, major depressive disorder, trichotillomania, suicide and depression

## Abstract

Polycystic ovary syndrome (PCOS) is a common endocrine disorder that affects women of reproductive age. Women with PCOS may present with obesity, amenorrhea, oligomenorrhea, infertility, or androgenic features. Studies have shown the association of psychiatric disorders with endocrine disorders, including PCOS. We present a case of a PCOS patient with no prior psychiatric history who presented with suicidal ideation. We emphasize the importance of screening women for PCOS when presented with mental disorders through this clinical case. It is crucial to rule out any medical causes in patients predominantly presented with psychiatric symptoms. We highlight the possible role of bupropion and naltrexone in managing PCOS symptoms, depression with suicidal ideation, and trichotillomania through this unique and rare case.

## Introduction

Nearly 16 million individuals in the United States are afflicted with depression annually [1]. The diagnosis of depression is ubiquitous and not restricted to age or ethnicity. There are numerous prolonged symptoms associated with depression, such as lethargy, insomnia, irritability, and inability to concentrate. Even though the prevalence of depression is high, the actual cause of depression is multifactorial. The fifth edition of the Diagnostic and Statistical Manual of Mental Disorders (DSM-5) notes several factors that influence an individual's risk of becoming depressed, including having a relative with depression, medical problems, use of illicit substances, or certain medications [2]. Of individuals with one physical disorder, 9.3%-18% had depression compared to those without any physical disorders, where only 3.2% of participants had depression [3]. Patients with PCOS have a higher risk of mental health disorders. One of the illnesses commonly comorbid with depression is polycystic ovary syndrome (PCOS) [4]. A study found that 40% of women with PCOS were diagnosed with depression, 11.6% had anxiety disorders, and 23.3% had binge-eating disorder. A comparison of the rates of mental disorder between patients with PCOS and without PCOS found that women with PCOS had significantly higher rates of depression [4]. 

Polycystic ovary syndrome is a condition in which women of reproductive age have abnormal menstrual periods or higher androgen levels. Women with PCOS usually exhibit symptoms of irregular periods, excess androgens, or polycystic ovaries [5]. About five million women of reproductive age are affected by PCOS in the United States, consisting of 6%-12% of the population. Despite its high prevalence in the population, the exact cause behind PCOS is still unknown. However, insulin resistance is a common factor of PCOS, which influences the increase in androgen hormones. The Center for Disease Control (CDC) found that more than half of the patients diagnosed with PCOS are also diagnosed with Type 2 diabetes [6]. There is an enormous economic burden associated with PCOS and depression. The overall economic burden of diagnosing and treating PCOS and its related symptoms is $4.35 billion annually in the United States [7]. The financial burden of depression is approximately $210.5 billion per year [4]. 

Given the high comorbidity of depression, there is a poor quality of life associated with PCOS. One study utilized a modified PCOS Questionnaire, which tested the seven categories of emotional imbalances, infertility, abnormal menses, menstrual predictability, hirsutism, weight fluctuations, and acne, to investigate the quality of life of women with PCOS. The study revealed women with PCOS scored lower in all of the measured categories of the modified PCOS, and weight was the main factor correlated with the patients' consistency in taking anti-androgen medication [8]. In fact, weight gain was identified as the primary agent in the relationship between PCOS and poorer quality of life. The poorer quality of life commonly seen in women with PCOS is of grave concern due to the large number of suicide attempts made by women with PCOS, which is seven times higher than that of women without PCOS [9]. 

Glintborg and Andersen found PCOS patients were 1.6 times higher by the time of their diagnosis on the Charlson Comorbidity Index (CCI), which helps estimate the risk of mortality within a year. The higher CCI in women with PCOS is correlated with increased cardiometabolic and psychiatric morbidity [10]. The severity of the risks associated with PCOS makes it paramount to find correct medications to reduce PCOS symptoms. Weight fluctuations have previously been identified as the leading cause of poorer life quality in women with PCOS [9]. Therefore, treatments for PCOS should focus on weight gain associated with PCOS. Recently, 8 mg naltrexone and 90 mg bupropion were combined as a slow-release pill to aid in weight loss. Additionally, it is noted that bupropion and naltrexone have a higher efficacy together than each drug treatment alone. While naltrexone does not treat mood disorders, bupropion is a well-known antidepressant. Therefore, the naltrexone-bupropion combination would work two-fold to manage weight and treat depression commonly comorbid with PCOS [11]. This case study highlights the clinical role of naltrexone and bupropion in managing PCOS patients with depression and suicidal ideation.

## Case presentation

A 25-year-old obese Asian Indian woman with no known past psychiatric history or family history was brought to the ED for the assessment due to her suicidal ideation. According to her family, the patient had never had suicidal ideation or attempts in the past. Her vital signs were within the normal range. The patient's body mass index (BMI) was 31, height was measured to be 162.5 cm, and she weighed 182 pounds (lb).

During the interview, it was found that the patient felt low, with no interest in any activities in the past year. Apart from her work-related commute, she stayed at home. Upon further interviewing, she admitted to having sleep disturbance and binge eating. She suffered from hirsutism since the age of 17 years, which has worsened over the years. The patient complained of "bear-like" hair growth all over her body, her urge to keep pulling her body and scalp hair. The patient said she sometimes would pull her perianal hair so hard that it would bleed. The patient also complained of oligomenorrhea and dysmenorrhea. The patient said she was tired of having to keep checking her facial hair several times daily since she was worried her beard would have grown towards the end of the day. Based on the patient's evaluation and presence of bald spots on the scalp and folliculitis on the stomach from hair-pulling, she was also diagnosed with trichotillomania, a hair-pulling disorder. The patient also suffered from adult acne. 

She was admitted to an inpatient facility due to suicidal ideation and the management of major depressive disorder (MDD). Laboratory workup of complete blood count, comprehensive metabolic panel, and urine analysis found no abnormalities. Also, toxicological tests were negative. An endocrinology consult was recommended, and further laboratory tests for PCOS were ordered due to the presence of hirsutism. The patient was diagnosed with PCOS, with her laboratory reports showing an elevated luteinizing hormone (LH) to follicle-stimulating hormone (FSH) ratio of 3:1. Her serum thyroid-stimulating hormone (TSH), prolactin, androstenedione, testosterone, fasting blood glucose, estradiol, and sex hormone-binding globulin levels were in the normal range. 

The patient was started on bupropion and was stabilized on bupropion extended-release (XL) 300 mg once a day and naltrexone 50 mg once daily. She was started on drospirenone/ethinyl estradiol 3 mg/20 mg once a day to help with hirsutism and acne. Spironolactone 50 mg twice daily once in the morning was prescribed. Tretinoin gel 0.025% once at bedtime was started for acne. Cognitive-behavioral therapy was also started. The patient was discharged after seven days on the above-listed medications and advised to lose weight.

After three months, the patient had lost 17 lb and had no suicidal ideation in the follow-up visit. The patient did not complain of any other psychiatric symptoms, including depression. The patient declined urges of hair-pulling. The patient noticed an improvement in the acne problem. At the six-month follow-up visit, the patient had lost 33 lb, no symptoms of depression, binge eating, and trichotillomania. Naltrexone was discontinued. Bupropion was tapered down to 150 mg XL once a day and spironolactone reduced to 25 mg twice a day. The patient was continued on drospirenone/ethinyl estradiol 3 mg/20 mg once a day for menstruation, hirsutism, and acne.

## Discussion

Polycystic ovary syndrome is a hormonal disorder that affects 5%-10% of all women. The symptoms include hirsutism, acne, lack of ovulation, and irregular, absent, or heavy menstrual bleeding. The pathogenesis of PCOS is thought to be due to multiple factors involving genetics and the environment, including obesity, insulin resistance, and exposure to excess androgens [12]. The implication of obesity in depression is well known in women for the general population, and approximately two-thirds of women with PCOS are obese. There are many propositions for psychiatric comorbidities in PCOS patients, but the underlying association is not entirely understood [13]. A strong link has been found between the severity of depression and overall dissatisfaction with physical appearance in women with PCOS. It also reported lower self-esteem and a greater fear of negative appearance evaluation in women with PCOS. Hyperandrogenism and acne are associated with body dissatisfaction, whereas hirsutism and BMI affect self-esteem and body satisfaction and lead to a fear of negative appearance [12]. 

In a study, the mean (± standard deviation (SD)) age of the total sample (n = 88) was 22.26 years (SD = 3.55). The participants were university students (75%), single (88.6%), and unemployed (90.9%). The study found that the prevalence rate for all comorbid psychiatric disorders in the sample was 50% (n= 44). Major depressive disorder (33%) was the most common psychiatric disorder in this study, followed by generalized anxiety disorder (GAD) (13.6%) and binge-eating disorder (6.8%). Other psychiatric conditions that were noted were any mood disorder (35%), bipolar disorder-II (2.3%), any anxiety disorder (19%), social phobia (2.3%), panic disorder (2.3%), obsessive-compulsive disorder (1.1%), any eating disorder (9%), and bulimia nervosa (2.3%). Among the patients diagnosed with PCOS and psychiatric illness, 12 (13.6%) met the criteria for at least two psychiatric disorders. In these women, eight out of 12 had MDD and GAD; four out of 12 had MDD and binge-eating disorder [14]. The results showed higher rates of psychiatric comorbidities in women with concomitant PCOS. In the published work, the prevalence of psychiatric disorders in women with PCOS has been reported to range between 57% and 67% [12].

The association of elevated androgen levels and depression explains women with PCOS being at higher risk for mood disorders. Rasgon and Elman found that patients with PCOS were less depressed when given oral contraceptive pills (OCP) than those not being treated with OCPs. These results indicate a relationship between depression and hormonal factors in women with PCOS [15]. Abnormal eating patterns, specifically binge-purge habits and bulimia nervosa, are associated with PCOS [12]. Physicians should be aware of some of the psychiatric comorbidities when treating PCOS patients; they are MDD, GAD, and binge-eating disorder. The patient's self-esteem and female identity may be damaged by PCOS' clinical sequelae of obesity, acne, hormonal disturbances, hirsutism, infertility, and psychological distress. Hirsutism develops due to elevated androgen levels and is theorized to lead to the resultant trichotillomania in patients [16]. A study by Weiner et al. concluded that women with PCOS and levels of free testosterone (FT) between 10 and 26 pg/mL were more depressed than women without PCOS and FT<10 pg/mL, and women with PCOS and FT>26 pg/mL [17]. Central serotonin (5-HT) activity is affected by insulin levels and 5-HT system disruption that causes depression might also affect peripheral insulin sensitivity leading to diabetes mellitus and obesity. Infertility is another common issue facing women with PCOS, possibly contributing to depression and anxiety [16]. 

The prescribed and postulated treatments for depression in PCOS include; corticotropin-releasing hormone (CRH) antagonists, glucocorticoid receptor (GR) antagonists, mineralocorticoid receptor (MR) antagonists, and metformin. CRH antagonists have been suggested as possible antidepressants for PCOS. Treatment with CRH antagonists like ketoconazole and metyrapone has shown a decline in depression and anxiety scores. However, these trials do not reveal if these agents treat depression symptoms or if the affective symptoms will recur after long-term administration. Mifepristone, a glucocorticoid receptor (GR) antagonist, has been implicated to be useful in depressed patients with increased basal cortisol levels. Mifepristone may be useful for treating psychotic depression, regardless of its side effect of infertility in PCOS [15]. 

Spironolactone, generally used to treat hypertension, is an mineralocorticoid (MR) antagonist that decreases insulin resistance and fasting insulin levels in women with PCOS. Spironolactone also reduces androgen production, which directly decreases some of the androgenic symptoms of PCOS. Insulin resistance has been theorized as the common pathway in the pathogenesis of PCOS and depression. Therefore, treatment with spironolactone may have some effect as an antidepressant in women with depression and PCOS [15]. Insulin allows the tryptophan influx into the brain, which contributes to 5-HT synthesis. Therefore, medications that increase central 5-HT synthesis may help treat depression; these medications include metformin and alpha-lipoic acid. Naltrexone blocks the opioid receptor site, which is also known to stimulate the appetite. Animal studies have shown that opioids injections centrally or peripherally stimulate feeding in mammals. Compared to placebo, naltrexone, an opioid antagonist, led to decreased daily caloric intake, and these reductions were not dependent on the dose [18]. 

Bupropion acts by increasing the levels of centrally available dopamine and norepinephrine by blocking their re-uptake. Recent studies have suggested that a decrease in dopamine concentration within the synapse of the hypothalamic neurons induces obesity, and it is this pharmacological mechanism that bupropion exploits to treat both the associated obesity and depression in PCOS [18]. Additional animal studies have shown that mice, which are norepinephrine-deficient, demonstrate a slight increase in appetite. Bupropion and naltrexone help address the psychological healthcare interventions in women with PCOS [18]. 

In a major phase III clinical trial, a combination of naltrexone and bupropion resulted in a significant weight loss of approximately 6% from their baseline weight [18]. The combination also improved glycemic control among people with Type 2 diabetes. Common side effects of naltrexone and bupropion use are nausea, constipation, dizziness, and dry mouth. Bupropion has greater efficacy as monotherapy, but both bupropion and naltrexone have been shown to promote weight loss in obese patients. Naltrexone further enhances the effects of bupropion, which in combination has the potential for additional weight loss compared to monotherapy [18-19]. PCOS management requires a multidisciplinary approach due to its complexity and heterogeneity. Early detection of depression with PCOS can help reduce the potential abuse of alcohol, tobacco, and other drugs that patients may use to mitigate their symptoms briefly [16].

## Conclusions

Polycystic ovary syndrome is a hormonal disorder of the reproductive age. Psychiatric disorders, especially depression, is often associated with PCOS. Studies have shown the adverse impact on the quality of life amongst women with PCOS due to psychiatric symptoms and higher rates of suicides compared to women without PCOS. All clinicians need to evaluate patients presenting with psychiatric symptoms for underlying PCOS thoroughly for timely and appropriate interventions. Weight gain, binge eating, hirsutism, and trichotillomania associated with PCOS have adversely impacted mental disorders. The possible role of bupropion and naltrexone is essential to study further in the management of PCOS with depression. We suggest multidisciplinary interventions to ensure proper medical and psychiatric care.
